# *Acinetobacter* Infections among Adult Patients in Qatar: A 2-Year Hospital-Based Study

**DOI:** 10.1155/2016/6873689

**Published:** 2016-06-28

**Authors:** Musaed Saad Al Samawi, Fahmi Yousef Khan, Yasser Eldeeb, Muna Almaslamani, Abdullatif Alkhal, Hussam Alsoub, Wissam Ghadban, Faraj Howady, Samar Hashim

**Affiliations:** ^1^Infectious Diseases Division, Department of Medicine, Al Khor Hospital, Al Khor, Qatar; ^2^Department of Medicine, Hamad General Hospital, Doha, Qatar; ^3^Infectious Diseases Division, Department of Medicine, Hamad General Hospital, Doha, Qatar; ^4^Department of Medicine, Al Khor Hospital, Al Khor, Qatar

## Abstract

This retrospective study was conducted at Hamad General Hospital, Qatar, to describe the demographic data, clinical features underlying diseases, antimicrobial susceptibility, and outcome of* A. baumannii* infection. It involved all adult patients 15 years of age or older who were managed at Hamad General Hospital for* A. baumannii* infection from January 1, 2012, to December 31, 2013. We identified a total of 239 patients with* A. baumannii* infection, of which 182 (76.2%) were males. The mean age was 49.10 ± 19.57 years. The majority of the episodes (25.1%) occurred in elderly patients (≥65 years) and the most commonly identified site of* A*.* baumannii* infection was the respiratory tract, 117 (48.9%). Most episodes of infection, 231 (96.7%), were hospital-acquired and high rate of nosocomial infections occurred in the medical intensive care unit, 66 (28.6%). All patients had underlying medical conditions. Maximum resistance was seen to cefotaxime, 147 (58.3%), and minimum resistance was seen to colistin, 2 (1.4%). Of the 239 isolates, 102 (42.7%) were susceptible and 137 (57.3%) were multidrug-resistant. The in-hospital mortality in our study was 31%. Male gender, multidrug resistance, and septic shock were found to be independent mortality predictors.

## 1. Introduction

*Acinetobacter baumannii* is an aerobic Gram-negative coccobacillus that has emerged as an important opportunistic pathogen, especially among debilitated patients, and a common cause of hospital-acquired infections, such as bacteremia, pneumonia, meningitis, urinary tract infection, and wound infection, especially in intensive care units [1, 2].* A. baumannii* is also a cause of community-acquired infections in many countries [3, 4]. The epidemic potential and the clinical severity of* A. baumannii* infections are related to resistance of the isolates to most classes of antibiotics through multiple mechanisms [1, 2].

In Qatar, the epidemiology of* A. baumannii* infection has not been studied in detail before. Only few reports have been published [5, 6]. The aim of this study was to describe the demographic data, clinical features, underlying diseases, antimicrobial susceptibility, and outcome of* A. baumannii* infection at Hamad General Hospital, Qatar.

## 2. Materials and Methods

### 2.1. Design and Setting

This hospital-based retrospective study was conducted at Hamad General Hospital. It involved all adult patients 15 years of age or older who were managed at Hamad General Hospital for* A. baumannii* infection from January 1, 2012, to December 31, 2013.

### 2.2. Case Definition

Hospital-acquired infection was defined as occurrence of infection 48 hours or more after hospital admission or within 48 h after discharge or if it was preceded by an invasive procedure [6]. Community-acquired infection was considered if the positive culture was obtained within the first 48 h of hospitalization and the patient was not hospitalized in the preceding month [6]. Empirical antimicrobial therapy was defined as treatment that included at least one antibiotic and that was started no later than 24 h after the blood sample for culture had been drawn. This therapy was deemed inadequate if the antibiotics were administered after 24 h of blood sample extraction and/or when the dosage, route, and duration of treatment were not in accordance with current medical standards [7].* A. baumannii* infection was defined as any patient who had* A. baumannii* isolated from any site in conjunction with a compatible clinical picture warranting treatment with antibiotics effective against* A. baumannii* [8]. Disease severity was estimated by the presence of sepsis or septic shock, which was defined according to the American College of Chest Physicians/Society of Critical Care Medicine Consensus Conference. Sepsis was defined as the systemic inflammatory response (SIRS) to infection and septic shock was defined as sepsis- (SIRS-) induced hypotension despite adequate fluid resuscitation along with presence of perfusion abnormalities that may include, but are not limited to, lactic acidosis, oliguria, or an acute alteration in mental status. Patients receiving inotropic or vasopressor agents may no longer be hypotensive by the time they manifest hypoperfusion abnormalities or organ dysfunction, yet they would still be considered having septic (SIRS) shock [9].* A. baumannii* isolates were considered multidrug resistance (MDR) if they exhibit resistance to carbapenem or resistance to at least one agent in three or more antibiotic classes [10]. The primary outcome was crude in-hospital mortality, which was defined as all causes of death during admission (i.e., infection-related mortality and mortality due to other causes).

*Antibiotic Susceptibility Testing*. After* A. baumannii* had been isolated from a range of clinical specimens, all isolates underwent susceptibility testing by the broth microdilution method (BD Phoenix; Becton Dickinson, Franklin Lakes, NJ, USA). The susceptibility breakpoints are defined by the Clinical and Laboratory Standards Institute [11].

### 2.3. Case Identification and Data Collection

All cases were identified from the hospital's microbiology records. The files and the electronic records of the patients were reviewed to identify infections and to retrieve the following data: demographic information; underlying conditions such as prior MRSA colonization; type of infection (community-acquired or hospital-acquired); antimicrobial susceptibility; and the outcome.

### 2.4. Data Analysis

Quantitative variables are expressed as mean ± standard deviations. In identifying the independent risk factors for in-hospital mortality, logistic regression model was used for univariate and multivariate analysis. The following variables were analyzed: age, sex, nationality, clinical picture, presence of other medical conditions, multidrug resistance isolates, and severity of infection. Univariate logistic regression was performed to determine the probable predictors of mortality among patients with* A. baumannii* infection. All potential risk factors significant at the 0.1 level in the univariate analysis were entered in the multiple logistic regression to identify the independent predictors of mortality at *P* < 0.05. The data were analyzed with SPSS software (v 17; IBM Corp., Armonk, NY, USA).

### 2.5. Research Committee Approval

As the study was retrospective, a waiver of informed consent was obtained from the research committee at Hamad Medical Corporation.

## 3. Results

During the period of study, a total of 372 consecutive* A. baumannii* isolates were collected by the microbiology department at Hamad General Hospital, which represent 2.8% (372/13286) of total isolates and 3.6% (372/10333) of Gram-negative isolates in our hospital. Based on evaluation of clinical charts, 239/372 (64.3%) isolates were classified as infection and 133/372 (35.7%) were considered colonizers. Of the 239 patients with* Acinetobacter* infection, 182 (76.2%) were males and 166 (66.5%) were non-Qataris. The mean age (± standard deviation (SD)) was 49.10 ± 19.57 years (range: 14–99 years).  Table 1 illustrates the demographic and clinical data of the 239 patients. Sixty episodes (25.1%) occurred in elderly patients (≥65 years).  Table 2 describes the distribution of the 239 infections among different age groups.

The most commonly identified sites of* A*.* baumannii* infection were the respiratory tract, 117 (48.9%), followed by urinary tract, 42 (17.6%), and blood, 40 (16.7%). The sites of* A*.* baumannii* infection are listed in Table 3. Most episodes of infection, 231/239 (96.7%), were hospital-acquired and high rate of nosocomial infections occurred in the medical intensive care unit (MICU), 66/231 (28.6%) (Tables 1 and 4).

All patients had an underlying medical conditions; 156 (65.3%) were on mechanical ventilation and 148 (61.9%) were on enteral feeding (Table 1). Maximum resistance was seen to cefotaxime, 147 (58.3%), and minimum resistance was seen to colistin, 2 (0.8%).

Of the 239 isolates, 102 (42.7%) were susceptible and 137 (57.3%) were multidrug-resistant (MDR). From these 137 MDR isolates, 2/137 (1.4%) showed resistance to colistin and 5/137 (3.6%) were resistant to tigecycline. All community-acquired isolates were susceptible except for one. The* in vitro* activity of individual antimicrobial agents against* A*.* baumannii* clinical isolates is summarized in Table 5.

The crude in-hospital mortality in our study was 65/239 (31%). By the univariate analysis, the following variables were associated with increased in-hospital mortality: old age (>65 year), male gender, pneumonia as underlying disease, multidrug resistance, septic shock, and inadequate therapy. Only male gender, multidrug resistance, and septic shock were found to be independent predictors of in-hospital mortality by multivariate logistic regression analysis (Table 6).

## 4. Discussion

To our knowledge this is the first report detailing the demographic data, clinical features, underlying diseases, antimicrobial susceptibility, and outcome of* A. baumannii* infection in Qatar.

As noted, most of our findings are consistent with several reports worldwide; for example,* A. baumannii* infection occurred in the elderly, mainly male patients; the vast majority of cases were hospital-acquired, affecting critically ill patients, especially those admitted into the intensive care units [1–3, 12–15].

Our results are, however, different from other studies in antimicrobial susceptibility pattern of* A. baumannii* isolates. These findings are the most relevant issue in this study and deserve special attention especially resistance pattern of the isolates towards carbapenems. The emergence of multidrug-resistant (MDR) isolates significantly limits effective therapeutic options. Therefore, monitoring antibiotic resistance patterns of this organism over time may provide useful information regarding its treatment policy. In the present study the prevalence of MDR among* A. baumannii* isolates accounted for 57.3% of the total, and the resistance to carbapenems represented 45.6% of total cases. These findings are less than those reported in India, Pakistan, Iran, Libya, Saudi Arabia, Greece, Turkey, Spain, and Italy [12, 14–24]. This findings are, however, alarming, suggesting the emergence of carbapenem-resistant* A. baumannii* and demonstrating the potential for this pathogen to become a major cause of nosocomial infections in Qatar; in addition, since the prevalence of MDR* A. baumannii* in Qatar was not determined before, our data can be used as a reference to assess any increase in the prevalence of resistant* Acinetobacter* in the future.

It has become increasingly clear that resistance development in* A. baumannii* is multifactorial and could be mediated by several mechanisms including (i) decreased permeability of antibiotics due to porin loss, (ii) alterations in penicillin-binding proteins, (iii) efflux pump and integrons, and (iv) hydrolysis of *β*-lactams by *β*-lactamases encoded by either plasmids or chromosome [25–27]. As noted, our study was descriptive, and there was data lacking on molecular epidemiology. Thus, we are unable to investigate the molecular basis of resistance among our isolates.

Other finding in our study which deserves attention is susceptibility pattern of* A. baumannii* isolates towards colistin and tigecycline. Literature data on the* in vitro* activity of tigecycline and colistin against* A. baumannii* shows variable susceptibility. Concerning tigecycline, 3.6% of our isolates were nonsusceptible, which is less than the proportions from Italy, India, and Taiwan [15, 28, 29] but similar to those reported in Turkey, Greece, and Thailand [30–32]. On the other hand, with few exceptions [33], recent reports show high antimicrobial activities of colistin against* A. baumannii* isolates, in agreement with our findings [32, 34]. These data suggest that tigecycline and colistin can be used effectively against MDR* A. baumannii* isolates in our hospital; however resistance rates should be monitored closely.

The crude mortality of* A. baumannii* infection varies ranging from 26 to 68% [13]. In our study, in-hospital mortality was 31% (65/239) which falls within the abovementioned range. Although drug resistance of* A. baumannii* is a recognized problem, the effect of MDR* A. baumannii* infections on patient outcomes remains controversial [35]. By univariate analysis, we recognized several clinical variables as probable predictors of in-hospital mortality; only male gender, multidrug resistance, and septic shock were found to be independent predictors of in-hospital mortality by multivariate logistic regression analysis.

This study has several limitations that need to be considered when interpreting these data. First, the study was retrospective. Second, it is small and hospital-based. Third, the study period was short. Fourth, data on molecular epidemiology of* A. baumannii* are lacking.

Despite these limitations, this study allowed us to reach important conclusions regarding resistance pattern among* A. baumannii* isolates in our hospital, which has an implication for future work. Accordingly, we suggest conducting large-scale long-period prospective studies on molecular epidemiology of* A. baumannii* with emphasis on drug resistance, in order to determine the population structure and their antimicrobial resistance patterns.

In conclusion, our findings revealed that* A. baumannii* is an important human pathogen that is gradually gaining more attention as a public health threat in Qatar. The emergence of carbapenem resistance means that there is a need to look for alternatives such as colistin and tigecycline, which retained greatest activity against the* A. baumannii* isolates. Infection with MDR* A. baumannii* is independently associated with high mortality, emphasizing the need for aggressive infection control strategies to prevent MDR* Acinetobacter* infection and its adverse effects on hospitalized patients.

## Figures and Tables

**Table 1:** Demographic and clinical data of the 239 patients involved in this study.

Variable	Number (%)/mean ± SD
*Gender*	
M	182 (76.2%)
F	57 (23.8%)

*Age* (mean ± SD)	49.10 ± 19.57 years

*Nationality*	
Qatari	73 (30.5%)
Non-Qatari	166 (66.5%)

*Underlying medical conditions*	
Prior MRSA colonization	18 (7.5%)
ICU stay	43 (17.9%)
Bed-ridden	51 (21.3%)
Prior *Acinetobacter*	15 (6.3%)
Tracheostomy	76 (31.7%)
Mechanical ventilation	156 (65.3%)
Pneumonia	108 (45.2%)
Diabetes mellitus	86 (35.9%)
Malignancy	27 (11.3%)
Recent surgery	86 (35.9%)
Invasive procedure	147 (61.5%)
Hemodialysis	43 (17.9%)
Previous antibiotic usage	105 (43.9%)
Enteral feeding	148 (61.9%)

*Infection setting*	
Hospital-acquired	231 (96.7%)
Community-acquired	8 (3.3%)

*Severity *	
Without sepsis	166 (69.4%)
Sepsis	3 (1.3%)
Septic shock	70 (29.3%)

*Adequacy of treatment*	
Adequate	90 (37.7%)
Inadequate	149 (26.3%)

*Outcome*	
Survived	174 (69%)
Died	65 (31%)

**Table 2:** Number of isolates among different age groups.

Age groups	Number of isolates (%)
15–24	28 (11.7%)
25–34	43 (18.0%)
35–44	31 (13.0)
45–54	38 (15.9%)
55–64	39 (16.3%)
≥65	60 (25.1%)

*Total*	239 (100%)

**Table 3:** Sites of infection.

Primary infection	Number (%)/
Blood	40 (16.7%)
Respiratory tract	117 (48.9%)
Urine	42 (17.6%)
Wound swab	25 (10.5%)
Tissue biopsy	5 (2.1%)
Abdomen drain fluid	4 (1.7%)
Peritoneal fluid	4 (1.7%)
CSF	1 (0.4%)
Synovial fluid	1 (0.4%)

*Total*	239 (100%)

**Table 4:** Hospital-acquired isolates in different wards.

Ward	Number of isolates
Medical ICU	66 (28.6%)
Trauma ICU	64 (27.7%)
Surgical ICU	22 (9.5%)
Medical ward	40 (17.3%)
Surgical ward	39 (16.9%)

*Total*	231 (100%)

**Table 5:** Drug resistance of *A. baumannii*.

Drug	Drug resistance
Amikacin	82 (32.5%)
Gentamicin	116 (46.1%)
Ceftazidime	75 (29.8%)
Ceftriaxone	95 (37.7%)
Cefotaxime	147 (58.3%)
Septrin	94 (37.3%)
Cefepime	115 (45.6%)
Colistin	2/137 (1.4%)
Meropenem	115 (45.6%)
Imipenem	89 (35.1%)
Tigecycline	5/137 (3.6%)
Piperacillin/tazobactam	126 (50%)
Ciprofloxacin	118 (46.8%)
Levofloxacin	60 (33.8%)

**Table 6:** Results of multivariate analysis of predictors of in-hospital mortality.

Variable	Adjusted odds ratio (95% CI)	*P* value
Male gender	2.5 (1.33–4.62)	0.004
Multidrug resistance	2.2 (1.19–3.94)	0.01
Septic shock	0.26 (0.25–0.72)	0.02
